# The Dental Profession and Its Appropriate Work

**Published:** 1857-03

**Authors:** Geo. Watt


### THE DENTAL PROFESSION AND ITS APPROPRIATE WORK.

[A Valedictory Address to the Graduates of the Ohio College of Dental Surgery—Session of 1856–'57.

BY GEO. WATT.

GENTLEMEN:—Day after day for months past, it has been our privilege to meet you in the halls of this institution, and to address you on the subjects respectively assigned us. We need not now tell you that we have regarded this as a happy privilege, nor allude to the fact, that no circumstance has transpired during the period of our association calculated to mar the happiness of our intercourse. Nor would it at all become us to refer to the manner in which we have severally discharged our relative duties, we as teachers, and you as pupils; for, of our efforts, you are better able to judge than we can be, and the evidence of faithfulness on your part is already in your hands, in a visible and tangible form, and may be "seen and read of all men." But terrestrial associations are not permanent, and earthly ties are soon broken. The relations which we have sustained toward each other, for a lapse of time are now at an end, and you go from us. But still, before you go, we must be allowed to offer, and you to receive an additional evidence of our interest in you, and our desire for your future success. As the youth departs from the paternal mansion to engage in the battle of life, the parents bid farewell again and again, painfully prolonging the parting scene, and all to postpone, for a little, the period which snaps asunder the family tie. So we, though the tie is broken—though no longer our pupils—now offer you a few words of parting advice, as if to prolong that relation which has afforded us so much rational enjoyment. Nor will it surprise any, that in our remarks we forget all others, and address only you. The many friends who greet us by their presence will not feel slighted; for they are here on account of the interest felt for you in the position you occupy, and their thoughts being all toward you, and their feelings in sympathy with you, it would seem to them unnatural and out of place, should our thoughts wander from you to address them. And your late classmates will not feel that in addressing you we are neglecting them; for the members of the household who remain are never jealous of any attentions bestowed on the departing.

As this is the *commencement* of professional life with you, and as you are now fully initiated into the membership of the Dental Profession, we do not know that we can better occupy your time than by offering a few remarks respecting that profession and its appropriate work.

The dental profession came into existence at the call of suffering humanity, and is a necessary result of enlightened and refined civilization, acting in concert with an advanced state of medical science. Though a distinct profession, it belongs to the great family of medicine and surgery,—in short, it is a branch and part of the medical profession. In periods long gone by, when medical knowledge was confined to a few leading facts, contaminated with error and superstition, dental science, as now developed, was an impossibility. But when this knowledge became vastly extended by the energy of its votaries,—when all the sciences were explored and made tributary to its advancement,—when the sun of science had dissipated the darkness of superstition and dispelled the gloom of error, then the field of medical science was exhibited in its vast extent—too vast, indeed, for any individual mind to cultivate so as to develop all its resources for good. A division of labor was the natural consequence. When the farmer finds his fields too large, his grounds so extensive that light crops are the only rewards of his extended toil, and this too, from the fact, that when his labors are diffused over the entire domain, each part receives but a partial and imperfect cultivation, if endowed with good judgment, he limits his efforts to a part of the tract, and, by the concentration of his energies on this, he develops all its resources, and his toil is requited with abundant crops. So it has been with the medical profession. The field became so large that it must all be very imperfectly cultivated, or parts of it neglected, unless by a division of labor, and a harmonious assignment of the laborers, this could be prevented. It is on this principle that specialties in medicine are found, the profession of your choice being one of them, and the one, perhaps, just now the most prominent of all in the public mind.

The dental profession, we have said, is a *branch* of the medical. The figure is an impressive one. And who has not seen the branch, while attached to, and a part of the parent tree, languish and pine, for want of a due proportion of the nourishing sap which is appropriated by its more prominent and thrifty comrades? And how often have we seen the same small and stinted branch removed from the parental stem, and transplanted into appropriate soil, where it takes root, grows with the vigor of youth, becomes itself a tree, and stands in majesty and beauty the pride and peer of its parent?

Much like this is the history of dental science. While it was cultivated only in connection with general medicine it made but little progress. Dental disease was overlooked, if not disregarded, on account of the claims of diseases more serious and fatal. Operations on the teeth were ignored, because more important ones pre-occupied the mind of the surgeon. To corroborate these statements, it is only necessary to refer to the prominent agencies in the advancement of medical science, the schools and the literature of the profession. Look to either of these sources for information in regard to dental science, and the little you will find will barely repay the research. Indeed, while our *branch* was cultivated as a part of the *parent tree*, the real nature of the most prominent dental disease was not discovered, nor even suspected.

We do not intend by these remarks to reflect on the character of the medical profession, nor, in any degree, to charge its members with intentional neglect of the dental apparatus. Far from it. As well might we find fault with the farmer for harvesting his field crops, to the neglect of his kitchen garden. But we do, on account of this state of affairs, regard the existence of our specialty as an evident necessity. When our branch was removed from the parent profession, implanted in the soil of science, refreshed by the dews of art, and enlivened by the light of genius—when it sent forth its roots in the shape of dental societies, periodicals and colleges, then it grew and prospered "like a tree planted by the rivers of water," sending out its boughs unto the sea, and "its branches unto the river," and now it stands the companion of its parent in relieving the ills of suffering humanity, and restoring the charms of faded beauty.

The members of the parent profession, to a great extent thus relieved from the care and responsibility connected with dental disease, have the more time to devote to other morbid conditions, and thus, by a division of labor, the advantages are mutual and general; and the success of our attempt at

*standing alone* will, no doubt, tend to produce a still farther division, which, if properly made, we are convinced, will produce equally satisfactory results.

It would be well, gentlemen, for you, and for us all, to take a profitable hint from the figure just used. The implanted branch always truly reflects the character of the parent tree. If the one be an oak, the other never becomes an elm, and far less a bramble. So we, though now organized and acknowledged as a distinct profession, should faithfully reflect our parentage by acting, at all times, worthy of the great medical profession of which we claim to be a humble part. We should always look to the light of genuine science to guide us in cases of doubt and difficulty; and we should ever press onward and upward to that perfection which all should desire. And, in accordance with our genealogy, we must see that we act worthy of the liberal profession to which we belong. We must not permit it to degenerate from its inherited position to that of a mere mercenary trade. We must remember that we are soldiers enlisted in the cause of humanity, and that, therefore, empiricism is not only unworthy of us, but hostile to us, and is, indeed, the prominent foe which we are called to oppose.

Activity is the natural condition of man. We not only live in a busy world, but our world is a part of a busy universe. Our Creator, though he works either with or without *means*, never works, either in creation or providence, without motive. And as the dental profession has come into existence in subservience to his providence, we conclude it exists for a definite purpose—that it has a special and appropriate work. And as we believe the profession has not yet fulfilled all its mission, this work may be appropriately viewed in its progressive state. Examined in its several parts, we might consider the portion already done, that now in progress, and the part yet undone.

In considering that portion of the work already done, we will understand ourselves better by casually noticing the early condition of the profession, or perhaps the state of medical science during the formative period of our profession. It would seem, from the best evidence extant, that, as early as the days of Herodotus, medicine and surgery were distinct professions, and that the latter was subdivided—dental surgery being one of its divisions. In the palmy days of Greece and Rome, dental surgery was cultivated to some extent; but as the light of science had not yet dawned on the masses of mankind, a knowledge of our art was almost totally lost in the gloom of the "dark ages."

But, at the revival of letters, dental science again made its appearance, in connection with medicine, and was mainly, if not totally, cultivated in this connection till near the beginning of the present century. About this period several members of the medical profession, conscious of the negligence manifested toward this department of surgery, turned their attention to it with commendable zeal and earnestness. Their views and discoveries were widely disseminated, and, by the light thus afforded, the darkness was rendered so distinctly visible that its dissipation was felt to be a necessity. From this period dental surgery was cultivated, for a few years, partly as a separate branch, and partly in connection with the general profession, and was thus supported and led by the parental hand till able to walk alone.

When all things are duly considered, we must regard the mere working of its way into a separate state of existence as the greatest achievement of the dental profession. In its earliest existence, our young profession had much to contend with and many obstacles to overcome. The prejudice and suspicion incident to any innovation were not lacking in this case; and these were, doubtless, in some measure excited and rendered more virulent by the character and conduct of members. Though for a time pretension without merit may be apparently successful, yet in the end it must fail. And accordingly, many of those who attached themselves to the young profession from selfish and mercenary motives were soon estimated according to their true character, and were driven out by the force of public opinion. The proper formation and direction of this public opinion was no insignificant part of the work devolving on the worthy and legitimate members of our profession in an early day. Indeed, this part of the work is but very partially accomplished yet; and it will not be complete till the public mind is so enlightened as to render the tricks and impositions of the charlatan an impossibility.

In emerging from the darkness of neglect, it was little wonder if dental science found itself beclouded by the gloom of ignorance. No one who looks back through the advancement of science in any other department, will be surprised at a want of knowledge exhibited by the early dental surgeons. Their profession was in its infancy, their duties undefined, and their means of progress few and untried;—and besides, they were few in number, and lacked that confidence in each other, which is only developed by acquaintance and association. But, like children worthy of their parentage, they ransacked the fields of science, and brought their varied treasures into the store-house of their profession. Anatomy, physiology, pathology, chemistry, and natural philosophy were all made to contribute, and, guided by the light of these, our profession has progressed from the smallest beginnings to its present position.

As the dental profession came into existence, it felt the want of a literature of its own. The great diffusedness of dental knowledge through the text-books of medicine rendered its acquisition tedious and difficult. Hence, we soon find a number of treatises and essays devoted solely to dental surgery, answering more or less perfectly the purposes of text-books. The permanent literature of the profession was rapidly extended; and in due time large volumes, devoted to our department of surgery, were published and widely disseminated among the profession.

But the publication of standard works could not keep pace with the rapid progress of dental knowledge, and the periodical press was resorted to. The progress in this department was rapid. The "American Journal," the pioneer, was soon followed by others; and to this day there is, perhaps, no greater evidence of the vitality of the dental profession than its periodical literature.

But the light which began to be thus diffused only rendered the darkness more annoying—the taste for knowledge thus enjoyed only increased the thirst for more; and we soon find the more advanced and energetic members of the profession forming themselves into an association for mutual improvement in professional knowledge. Kindred associations were soon formed in other localities, till, finally, like our parent, we have local, state, and national associations, all doing much for the cause of progress.

A lack of thorough training in the various sciences pertaining to the profession was soon felt to be the great want. And as the uniform practice of enlightened communities bore testimony in favor of collegiate instruction, dental colleges became a professional necessity. These were organized, and we thus find our young profession in the enjoyment of all the means of improvement which are possessed by its parent, the medical profession.

The combined influences of these various agencies produced corresponding results. Each department of our profession, accordingly, made rapid progress. The pathology of the principal diseases of the teeth and adjacent parts is much better understood than formerly; and, of course, the treatment is more correct and reliable. And in the mechanical department of the profession, the progress is equally striking, both in utility and appearance. The unsightly ivory and the dead teeth are banished; and their places are filled by gems of the purest porcelain, surpassed only by the natural organs.

We have thus noticed briefly that part of the work already done. Need we tell you what our profession is now doing? Need we tell *you* that it is delivering old age from one of its greatest infirmities, and thus smoothing the passage of life's decline? that it is reproducing the faded charms of youth and beauty? that it is restoring to the unfortunate orator the musical intonations of his lost eloquence? that it is relieving humanity of one of its sharpest pangs? These are among the tamest of its triumphs. Diving deeper than all these, it is arresting the ravages of dental diseases, preventing their sad consequences, obviating the dire results of impaired and defective nutrition, thus lengthening out the span of man's pilgrimage, while adding much to his comfort and enjoyment by the way.

But it is useless to talk of the present. It is but the connecting link of the past and future—but the dividing point between two eternities.

Let us, then, for a moment, turn our attention to the future, and survey that part of the work yet to be accomplished. And here we would fain have the lengthened range of vision granted to the prophet on the mount; for we fear that much of our professional land of promise lies afar off—so far, indeed, that our carcasses will fall in the wilderness of effort that intervenes; yet when we fall, we trust that you who are younger, and others who are to follow in your footsteps, will press on toward the prize; "for there is yet much land to be possessed."

In a careful review and accurate estimate of the work that lies before us, there may be found much to discourage; yet it is better to take this view, and make this estimate at the outset, that the faint-hearted may be allowed, as in the army of Gideon, to turn back from the contest. Let us endeavor, then, to take a calm and deliberate look at the work before us.

Connected with that beautiful set of organs especially committed to our professional care, we find many evidences of the frailty of man. A want of harmony in the human constitution is usually developed by the earliest manifestations of the dental organs. The constitutional disturbances arising from dentition are many and aggravated. Some authors estimate that one-tenth, or even a sixth of the human race, die during this period; and all agree that the process of dentition has much to do in producing the morbid conditions so fatal in their effects. That this state of affairs results from the violation of the laws of health, none will deny. A portion of the work, then, that devolves on us is to ascertain these laws, and disseminate a knowledge of them, that consequences so serious may be avoided. When the cause is removed, the effect ceases. We must, then, in concert with the medical profession, search out the causes of difficult dentition, whether hereditary or incidental, and devise means for their removal, that the whole process may take place in accordance with physiological laws, and, consequently, without disturbance to the health.

But dental difficulties do not cease with the eruption of the organs. In many instances, the teeth decay as soon as they emerge through the gum; and but few persons arrive at maturity without losing some of those organs, and suffering the tortures of tooth-ache besides. You who are familiar with the admirable laws which the Creator has established for the formation and growth of the teeth, will agree that this is all wrong; and even the most casual observer, when he considers the bony hardness, and looks at the glassy enamel of the teeth, must conclude that they were made to last through life; when he sees them so beautifully adapted to the purposes of mastication, he must conclude that they were formed for *use* as long as a necessity for food should exist. This early decay exhibits a serious violation of the laws of health. It evidences that the teeth are imperfectly formed and developed, that the fluids naturally in contact with them are depraved, or that substances highly improper are permitted to come in contact with them. One of these propositions must be true, they all may be true. We are, then, called on to ascertain the precise nature and extent of the various influences brought to bear in the destruction of the dental organs. And if we find that hereditary predisposition exerts a malign influence, as doubtless we will, the public mind must be enlightened, and the habits of society reformed, till these organs no longer remind us that the iniquities of the fathers are visited upon the children. We must be able, from an examination of the constitution and habits of the patient, the state of the secretions, and the character of the decay in the tooth, to recognize the chemical qualities of the corroding agent in each and every case of dental caries. And, having ascertained these, we must know how to obviate its devastations and eradicate it from the system. This will require greatly increased knowledge in all the sciences pertaining to our profession—vastly increased light in all its departments; and when the triumph of dental science is complete, the arts of dentistry, operative and mechanical—both so important now—will almost cease to be needed.

Thus far, we have alluded only to our work as members of the dental profession; but our labor does not cease here, and must not be so restricted. While we are members of the profession, we are also members of society at large; and as membership always implies corresponding duties, we are required, not as a profession, but as individuals, to bear our part in the cares and toils of life. And, as we are proud to call ours a liberal profession, it becomes us, for our own credit, to devise liberal things. Society always expects more from the enlightened and the educated than from those whose opportunities and attainments are more limited; and this is right. "To whom men have committed much, of him will they ask the more," is the language of the Savior, while he applies the principle to his own government, in adjusting the final affairs of men. We have, then, to do our part, not only socially and morally; but we must contribute our portion to the general advancement of all useful knowledge.

But there is a limit to human knowledge. The finite mind can never approach, far less vie, with the Infinite. The science of our profession must, therefore, like other sciences, be but partially understood. In all cases of scientific search, whether we dig in the mines of knowledge or climb the hill of science,

> Each deep explored reveals a deeper depth—
> Each height attained displays a height beyond.

Antecedent to each effect is its cause, and behind this is that which caused it; and thus we are led back to the philosopher's great First Cause, which is none other than the Christian's God. This is the end of all science; and unless we are led in this direction, we have studied to little purpose.

But, gentlemen, it is folly for us to farther prolong this scene. The time has come for us to part. To part!—but, shall we meet again? And if so, where? and when? Shall it be in these halls a year hence, when your junior classmates are placed in the position now occupied by you? Shall it be in the dental society, or the national convention? Not likely in any of these. Ere another meeting, it is not improbable that some of us will go the way whence we will not return; but, if not, there is little probability that circumstances will be such as will enable us all to take part in any single assembly of our profession. There is, therefore, little probability of us all meeting here; but a convention is appointed; the time will be duly announced; we will all be called to attend; and, without any regard to circumstances, we will all be there. We have met here, and we part now. We will meet there, shall we part then?

But we must not detain. As members now of the dental profession, we welcome you to our ranks;—as pupils of this Institution, we bid you farewell.

